# A Nondestructive Detection Method for the Muti-Quality Attributes of Oats Using Near-Infrared Spectroscopy

**DOI:** 10.3390/foods13223560

**Published:** 2024-11-07

**Authors:** Linglei Li, Long Li, Guoyuan Gou, Lang Jia, Yonghu Zhang, Xiaogang Shen, Ruge Cao, Lili Wang

**Affiliations:** 1 College of Food Science and Engineering, Tianjin University of Science and Technology, Tianjin 300457, China; lll8880223@163.com (L.L.); gouguoyuan@163.com (G.G.); jl15135408007@163.com (L.J.); 2 Institute of Food Science and Technology, Chinese Academy of Agricultural Sciences, Beijing 100193, China; llzgnydx@163.com; 3 Shandong Engineering Research Center for Grain and Oil Deep Processing, Linyi 276699, China; zhangyonghu@chinayuhuang.com (Y.Z.); shenxiaogang@chinayuhuang.com (X.S.)

**Keywords:** oat, near-infrared spectroscopy, feature selection, nondestructive, portable near-infrared spectrometer

## Abstract

This study aimed to achieve a precise and non-destructive quantification of the amounts of total starch, protein, β-glucan, and fat in oats using near-infrared technology in conjunction with chemometrics methods. Eight preprocessing methods (SNV, MSC, Nor, DE, FD, SD, BC, SS) were employed to process the original spectra. Subsequently, the optimal PLS model was obtained by integrating feature wavelength selection algorithms (CARS, SPA, UVE, LAR). After SD-SPA, total starch reached its optimal state (*R*_p_^2^ = 0.768, *RMSEP* = 2.057). Protein achieved the best result after MSC-CARS (*R*_p_^2^ = 0.853, *RMSEP* = 1.142). β-glucan reached the optimal value after BC-SPA (*R*_p_^2^ = 0.759, *RMSEP* = 0.315). Fat achieved the optimal state after SS-SPA (*R*_p_^2^ = 0.903, *RMSEP* = 0.692). The research has shown the performance of the portable FT-NIR for a rapid and non-destructive quantification of nutritional components in oats, holding significant importance for quality control and quality assessment within the oat industry.

## 1. Introduction

Oats are an incredibly nutritious whole grain offering a multitude of health benefits. They are particularly rich in protein, which is essential for muscle development and repair. In addition, oats are a great source of healthy fats, especially unsaturated fatty acids, which play a significant role in maintaining heart health. Furthermore, oats contain a unique dietary fiber called β-glucan, which helps lower cholesterol, regulate blood sugar levels, and promote a healthy digestive system. Oats are also packed with various vitamins and minerals, including vitamin E, B-complex vitamins, iron, and zinc, all of which are vital for the proper functioning of the body [1]. With the growing emphasis on healthy eating, the demand for oat-based products in the market is increasing. As a result, companies in the industry have focused their efforts on oat product research and development to meet consumers’ demand for healthy food choices. However, the oat industry faces challenges in terms of selecting the right varieties and ensuring quality control due to the vast variety and differences in quality among oats [2]. As a result, it is of utmost importance to establish an efficient, convenient, and non-destructive oat testing technique. This would provide producers with a better means for carrying out variety selection and quality control, while also offering consumers accurate nutritional assessments and facilitating the selection of high-quality products.

Thus far, there have been studies employing reference oat composition testing methods such as high-performance liquid chromatography (HPLC), mass spectrometry (MS), and gas chromatography (GC) [3,4,5]. However, these conventional methods are time-consuming, resource-intensive, operationally complex, and destructive. In comparison, near-infrared spectroscopy (NIRS) technology offers advantages such as rapidity, accuracy, and non-destructiveness [6,7]. It has already found widespread applications in various fields such as agriculture, food, pharmaceuticals, and the environment [8,9,10]. Hence, the adoption of non-destructive NIRS technology for the detection of key nutritional components in oats will significantly enhance the efficiency and quality control of oat testing. Furthermore, it will optimize the oat production process, cater to market demands more effectively, and facilitate the development of the oat market.

Nevertheless, during the NIRS data collection phase, the data can be susceptible to external environmental factors, instrument discrepancies, and sample properties, which may result in challenges such as baseline drift, noise, and scattering within the spectra [11]. These interferences have the potential to compromise the quality and accuracy of the spectra. Hence, preprocessing the raw spectra is a fundamental step in spectroscopic analysis. Common spectral preprocessing methods include standard normal variate (SNV), multiplicative scatter correction (MSC), normalization (Nor), detrend (DE), derivatives, and smoothing [12]. Yang et al. (2024) employed several spectral preprocessing techniques, including SNV, MSC, baseline correction (BC), noise correction (NC), first derivatives (FDs), second derivatives (SDs), as well as the mathematical transformations of these derivatives, to preprocess the original spectra and combined these preprocessing methods with three modeling approaches: successive projections algorithm–multivariate linear regression (SPA-MLR), partial least squares regression (PLSR), and support vector regression (SVR), to achieve the near-infrared detection of the β-glucan content in oat grains [13]. Teixido-Orries et al. (2023) employed three preprocessing methods, specifically SNV, FD, and a combination of both, to preprocess the spectra. This approach facilitated the quantitative analysis and sample classification of T-2 and HT-2 toxin levels in oat samples [14]. Mahgoub et al. (2024) preprocessed the collected spectra using FD and MSC, combined with PCA, SIMCA, and OPLS-DA, to analyze and identify the adulterants in different kinds of oat flour. Notably, both the SIMCA and OPLS-DA models exhibited a remarkable sensitivity of 100% [15]. Thus, the precise selection and application of appropriate spectral preprocessing methods are pivotal steps to ensure the accuracy and reliability of spectral analysis results.

In this study, the selection of suitable preprocessing methods had been explored. Of particular significance was the utilization of four feature wavelength selection algorithms to identify the most representative and informative wavelengths from a wide array of options in the preprocessed spectral data. The selected algorithms were competitive adaptive reweighted sampling (CARS), the successive projections algorithm (SPA), uninformative variable elimination (UVE), and least angle regression (LAR). This approach simplified the data, improved modeling accuracy and stability, and enhanced our understanding of the spectral data characteristics. Previous studies have predominantly focused on preprocessing methods for spectral data. In contrast, this study achieved a comprehensive analysis and interpretation of spectral data by combining preprocessing techniques with feature wavelength algorithms. This approach provided crucial support for a deeper understanding of oat characteristics. Furthermore, the findings of this study offered a more reliable data foundation for future research, thereby driving advancements in related fields.

The objectives of this study were to (i) analyze the content of four nutritional components (total starch, protein, β-glucan, and fat) in oats using conventional chemical methods, (ii) use a portable handheld near-infrared spectrometers to capture the oat spectra, and (iii) build regression models using a combination of the spectra pretreatment and the wavelength selection algorithms.

## 2. Materials and Methods

### 2.1. Samples

A total of 149 oat samples from different provinces (Zhangjiakou, Inner Mongolia, Gansu, Shanxi, Yunnan, Jilin) were collected. After manual screening and removal of impurities, the oat grains were scanned by near-infrared spectroscopy and ground by a Huachen multifunctional pulverizer (HCP-100, Guangzhou, China). Subsequently, the oat flour was stored at −20 °C for subsequent chemical composition determination.

### 2.2. Portable NIR Spectrometer System

The NIR data were acquired using a NeoSpectra Scanner developed by Si-Ware Systems company (Menlo Park, CA, USA). This state-of-the-art device employs chip-based optical systems for spectral acquisition, comprising mainly of a calibration module (standard block and optical window) and a power source, controlled directly via Bluetooth by IOS or Android devices. The optical window of the spectrometer spans an extensive wavelength range from 1350 to 2500 nm, boasting a remarkable spectral resolution of 16 nm, and its sample coverage diameter can reach up to 10 mm, ensuring a robust spectral response. Additionally, the instrument was designed with portability in mind (measuring 178 × 91 × 62 mm and weighing 1 kg), featuring an IP65 casing that is robust and durable. It is powered by a long-lasting battery, with ample memory to store up to 999 spectra. Furthermore, the scanned data were transferred and stored in a smartphone’s memory in CSV format, and the spectrum data were transferred to a computer and converted into the MAT file format. Finally, subsequent data analysis was performed using MATLAB R2023a software (The Mathworks, Inc., Natick, MA, USA).

### 2.3. Chemical Analysis of Total Starch, Protein, β-Glucan and Fat

#### 2.3.1. Total Starch Determination

The total starch content was measured utilizing the Megazyme total starch assay kit (Megazyme Ireland, Bray Town, Ireland). A 100 mg sample was combined with 10 mL of sodium acetate buffer (pH 5.0). Following that, the α-Amylase (0.1 mL) was introduced into the sample tube, while the buffer (0.1 mL) was added to the blank group. The contents were then oscillated for 3 s. Following a slight loosening of the test tube lid, the mixture was subjected to heating in a boiling water bath for 2 min. The lid was securely sealed and the contents of the tube were thoroughly mixed and further heated for an additional 15 min (with thorough mixing every five min). The test tube was then placed in a 50 °C water bath for 5 min to equilibrate. Subsequently, the Amyloglucosidase (0.1 mL) was added to the sample tube, with 0.1 mL of buffer added to the blank tube. The mixture underwent a 50 °C water bath for 30 min, followed by removal and equilibration at room temperature for 10 min. The sample (2 mL) was transferred to a centrifuge tube and centrifuged at 16,020× *g* for 5 min. Next, 1 mL of the supernatant was mixed with 10 mL of buffer, and three 0.1 mL glucose standard solutions were added. A 1 mL solution was added to a 5 mL tube, with 3 mL of GOPOD added, and incubated in a 50 °C water bath for 20 min. The absorbance was measured at 510 nm using an enzyme labeling instrument (Chaneleon, Hidex, Turku, Finland). The total starch content was calculated according to Equation (1).

Total starch (%) = ∆A × *F* × 10.2 × *D*/*W* × 0.9 (1)

where ∆A represents the difference between the absorption value of the sample and the absorption value of the reaction blank, *F* = 100/Absorbance of glucose standard solution, *D* is the multiple of sample dilution (if the absorbance value is less than 0.1, the sample does not need to be diluted), and *W* indicates the weight of the sample on a wet basis (mg).

#### 2.3.2. Protein Determination

The protein content in the sample was assessed following the first step of the Kjeldahl method as outlined in GB 5009.5-2016 [16]. Proteins were decomposed under catalytic heating conditions, and the ammonia produced was reacted with sulfuric acid to produce ammonium sulfate. Alkaline distillation was employed to liberate the ammonia, which was absorbed by boric acid. The absorbed ammonia was then titrated using a standard sulfuric acid or hydrochloric acid solution. The nitrogen content was derived from the quantity of acid consumed, and was subsequently multiplied by a conversion factor to ascertain the protein content. The protein content was calculated using Formula (2).

(2) $$X=\frac{(V_{1}-V_{2})\times c\times 0.0140}{m\times V_{3}÷100}\times F\times 100$$

where *X* represents the protein content in the sample (g/100 g), $V_{1}$ is the volume of standard sulfuric acid or hydrochloric acid titrant consumed by the test solution (mL), $V_{2}$ is the volume of standard sulfuric acid or hydrochloric acid titrant consumed by the reagent blank (mL), $V_{3}$ is the volume of the neutralization solution extracted by suction (mL), 0.0140 is the mass of nitrogen equivalent to 1.0 mL of standard sulfuric acid or hydrochloric acid titrant (g), *m* is the mass of the sample (g), *c* represents the concentration of the standard sulfuric acid or hydrochloric acid titrant solution (mol/L), *F* is the conversion factor for nitrogen to protein, and the conversion factor is 100.

#### 2.3.3. β-Glucan Determination

As per NY/T 2006-2011 [17] guidelines, the determination of β-glucan was conducted using the Megazyme β-glucan assay kit (Megazyme Ireland, Bray Town, Ireland) [13]. For the pretreatment of enzymatic hydrolysis, samples weighing between 0.08008 and 0.1000 g were measured and mixed with 0.2 mL of ethanol (50%) solution. The mixture was dispersed by vortex mixing and then 4 mL of sodium phosphate buffer was added. After thorough shaking, the sample was heated for 1 min in a boiling water bath, followed by vigorous vortex mixing for several seconds and a 2 min incubation in a boiling water bath. For the enzymatic hydrolysis reaction, the test tube was placed in a 50 °C water bath for 5 min, then 0.2 mL of lichenase solution was added and vigorously shaken for a few seconds. The sample was kept in a 50 °C water bath for 60 min (with agitation every 15 min). Subsequently, the test tube was removed, 5 mL of 200 mmol/L sodium acetate buffer was added, mixed thoroughly, and allowed to cool at room temperature for 5–10 min before centrifugation for 10 min. The supernatant was collected. From the bottom of three test tubes, 0.1 mL of supernatant was extracted. Glucosidase solution was added to two test tubes, while 0.1 mL of 50 mmol/L sodium acetate buffer was added to the remaining test tube as the reaction blank. These test tubes were then incubated at 50 °C for 10 min. Following this, 3 mL of GOPOD was added to each test tube, which was then placed in a water bath at 50 °C for 20 min. After cooling the test tubes at room temperature, the absorbance value was measured at 510 nm, and the content was calculated using Equation (3).

β-glucan (%) = ∆A × *F* × 94 × 10^−6^ × 100/*W* × 0.9 (3)

where ∆A represents the difference between the absorption value of the sample and the absorption value of the reaction blank, *F* represents the conversion factor for translating absorbance values into μg glucose, *F* = 100 μg glucose/100 μg absorbance of glucose. The volume correction factor is 94, *W* is the weight of the sample on wet basis (g), and the dehydration conversion factor that transforms glucose into β-glucan is 0.9.

#### 2.3.4. Fat Determination

The fat content in oat samples was determined utilizing the Soxhlet extraction method specified in GB 5009.6-2016 [18]. Sample preparation: 2–5 g of the thoroughly mixed samples were weighed out, accurate to 0.001 g, and transferred into a filter paper tube; then, approximately 20 g of quartz sand was added, and the mixture was steam dried on a boiling water bath, then dried in a hot air oven at 100 ± 5 °C for 30 min, ground finely, and transferred into the filter paper tube. The evaporating dish and glass rod were wiped with ether-dipped cotton to remove any sample residue, and the cotton was placed into the filter paper tube. Extraction: The filter paper tube was placed in the extraction tube of the Soxhlet extractor and connected to the dried receiving bottle; anhydrous ether or petroleum ether were added to a total of two-thirds of the bottle’s volume, before being heated on a water bath to continuously reflux the solvent extraction (6–8 times/h), which typically took 6–10 h. Upon completion, a drop of the extract was collected with a ground glass rod; no oil stain on the rod indicates the completion of extraction. Weighing: The receiving bottle was removed, the anhydrous ether or petroleum ether were recovered, and the mixture was evaporated on a water bath when the remaining solvent in the bottle reached 1–2 mL, before being dried at 100 ± 5 °C for 1 h and cooled in a desiccator for 0.5 h before weighing. The above steps were repeated until a constant weight was achieved (until the difference between two weights did not exceed 2 mg). The fat content was calculated according to Formula (4).

(4) $$X=\frac{m_{1}-m_{0}}{m_{2}}\times 1$$

where *X* represents the fat content in the sample in grams per 100 g (g/100 g), $m_{1}$ is the mass of the receiving bottle and fat after reaching constant weight in grams, $m_{0}$ is the mass of the receiving bottle in grams, $m_{2}$ is the mass of the sample in grams, and the conversion factor is 100.

### 2.4. NIRS Data Acquisition

At room temperature, 300–400 g of oat grains were weighed and deposited into a tray measuring 10 × 20 cm, with a layer thickness of around 3 cm. Following this, a portable near-infrared spectrometer was employed, linked to a mobile application through Bluetooth. The device was initially calibrated by aligning the standard block with the optical window, and subsequently aligning the optical window with the sample for scanning. Five scans were conducted at varied locations on each sample to acquire comprehensive data. Ultimately, the gathered spectra were utilized for subsequent analysis.

### 2.5. Spectral Data Preprocessing

Spectral preprocessing is an essential part of NIR analysis and plays a crucial role in enhancing the quality of spectra [19]. By synthesizing findings from relevant fields and considering the characteristics of the experimental data, this study employed eight preprocessing methods: SNV, MSC, Nor, DE, FD, SD, BC, and Savitzky-Golay Smoothing (SS). SNV is utilized to eliminate differences in spectral intensity caused by factors such as light source intensity and transmittance in spectroscopic data. It enhances the analysis of spectral features, peak positions, peak intensities, and other information among samples [20]. MSC is employed to eliminate scattering effects in spectra, improving peak shape and resolution for clearer spectra [21]. Nor is used to remove differences in spectral intensity between different samples, facilitating better comparison and analysis of spectral data [22]. DE helps eliminate the influence of samples on their NIR spectra and correct baseline drift in diffuse reflectance [23]. The derivative method overcomes band overlap and enhances spectral features [24], while BC removes baseline drift in spectra to eliminate instrument drift and other non-sample related interferences [25], and SS is used to reduce noise and fluctuations in spectra, enhancing the quality of spectral signals. These methods significantly enhance the quality of spectral data, providing a solid foundation for subsequent analysis, modeling, and interferences [25].

### 2.6. Characteristic Wavelengths Selection

Characteristic wavelength typically refers to a specific wavelength within the NIRS that demonstrates notable absorption or reflection peaks for a particular substance or specific chemical component [26]. These feature wavelengths hold significant value in facilitating the identification and quantitative analysis of substances present in the samples. By selectively filtering the feature wavelengths, it becomes feasible to exclude superfluous spectral information, thereby diminishing the data’s dimensionality while bolstering the computational efficiency and predictive prowess of models. This methodology fosters a more refined capture of the chemical information embedded within the samples, consequently expediting and ensuring accurate analysis [27]. In this study, on the basis of optimal preprocessing, four feature wavelength selection algorithms were employed for extracting characteristic wavelengths from the full spectra of the samples. These algorithms include CARS, SPA, UVE, and LAR. Subsequently, Partial Least Squares (PLSs) models were separately built for total starch, protein, cellulose, and total phenols parameters. By comparing the model results, the best wavelength extraction method was chosen.

CARS is a feature wavelength selection algorithm that utilizes Monte Carlo sampling to randomly select a subset of samples from the original dataset, thus creating an initial sub-dataset. For each sub-dataset, a regression model was constructed using PLSs or other suitable regression methods. The weights of each variable in the model are computed, reflecting their contributions to the predictive performance of the model. Through a competitive learning mechanism, variables with low weights are gradually eliminated, while those with high weights are retained. This process is adaptive, meaning that in each step, the weights are recalculated based on the currently retained variables, and further selection is performed. This process is repeated until a preset number of iterations or other stopping conditions are met. CARS is effective in handling high-dimensional data, reducing the computational burden, and improving the accuracy of feature selection through its adaptive reweighting mechanism, dynamically adjusting the importance of variables [28,29,30,31].

SPA starts by selecting an initial variable from the dataset and projects each variable onto the remaining variables, calculating the resulting projected vectors. The variable with the maximum norm in the projected vectors is then chosen as the next variable for selection. This projection and selection process is repeated until a predetermined number of variables or other stopping conditions are met. SPA is computationally efficient and relatively simple, making it suitable for handling large-scale high-dimensional data. Through iterative projections, SPA effectively retains key information in the data, reduces data dimensionality, and improves the predictive performance of models [32,33,34].

The UVE algorithm utilizes Partial Least Squares (PLSs) regression or other appropriate regression methods to build an initial model on the original dataset. In order to assess the importance of each variable, the UVE method generates a set of pseudo-samples, typically by randomizing the response variable in the original dataset. Models identical to the original model are then constructed on these pseudo-samples. For each variable, the difference in the regression coefficients between the original model and the pseudo-models is computed, which is referred to as the reliability index. Based on this reliability index, variables with values significantly above a threshold are considered informative, while those with lower values are considered uninformative and are removed from the model. UVE effectively eliminates uninformative variables, simplifying the model and reducing the risk of overfitting. By removing uninformative variables, UVE helps improve the generalization ability of the model, allowing it to perform better on new data [35,36,37].

The LAR algorithm initializes all regression coefficients to zero. It selects the variable with the highest correlation to the response variable (target variable). LAR effectively performs feature selection as it only selects the most correlated variable at each step [38,39].

Therefore, the selection of characteristic wavelengths assumes a critical role in the analysis of near-infrared spectroscopy. It serves to heighten the efficiency and precision of the analysis, while concurrently fostering a more profound comprehension of the chemical information embedded within the samples.

### 2.7. Model Establishment Method

Partial Least Squares (PLSs) is a multivariate regression technique that utilizes an iterative process to extract components. These components are linear combinations of the original independent variables and are selected to maximize the correlation with the dependent variable [40]. PLSs enhances the predictive capability of the model by considering the relationship between the independent and dependent variables during model construction. By extracting a small number of components, PLS reduces the dimensionality of the independent variables, simplifying the complexity of the model and improving its interpretability [41]. Furthermore, PLSs effectively addresses the issue of multicollinearity among the independent variables [42]. Thus, considering the requirements of this study, PLS was chosen as the method to establish the regression model.

### 2.8. Model Evaluation Criteria

In this study, the coefficient of determination (*R*^2^) and root mean square error (*RMSE*) were employed as evaluation metrics to assess the model’s performance [43]. *R*^2^ evaluates the model’s ability to explain the observed data’s variance, with values ranging from 0 to 1. A higher value indicates a better fit of the model. On the other hand, *RMSE* measures the difference between predicted values and the actual observed values. A smaller *RMSE* indicates a lower prediction error, implying a more accurate prediction by the mode.

## 3. Results

### 3.1. Statistical Distribution of Total Starch, Protein, β-Glucan, and Fat Content in Oats

In this study, the sample set of 149 oat samples was divided into a calibration set and a prediction set using sample set partitioning based on the Joint X-Y distance (SPXY) algorithm, following a 4:1 ratio. The application of SPXY in sample set partitioning is widely used and has shown superior performance compared to random sampling (RS), the Kennard-Stone (KS) algorithm, the duplex algorithm, and other methods [44]. SPXY ensures a balanced representation of sample characteristics in both the calibration and prediction sets. By integrating spectral and physicochemical information about the samples, the SPXY algorithm effectively partitions the samples into the calibration and prediction sets, ensuring good representativeness of sample characteristics in both sets [45]. This provides a solid foundation for subsequent modeling and prediction to be carried out. Table 1 provided detailed statistical information on the content of total starch, protein, β-glucan, and fat in the calibration and prediction sets of oat samples.

### 3.2. Raw Spectral Analysis of Oat Samples

**Figure 1:**
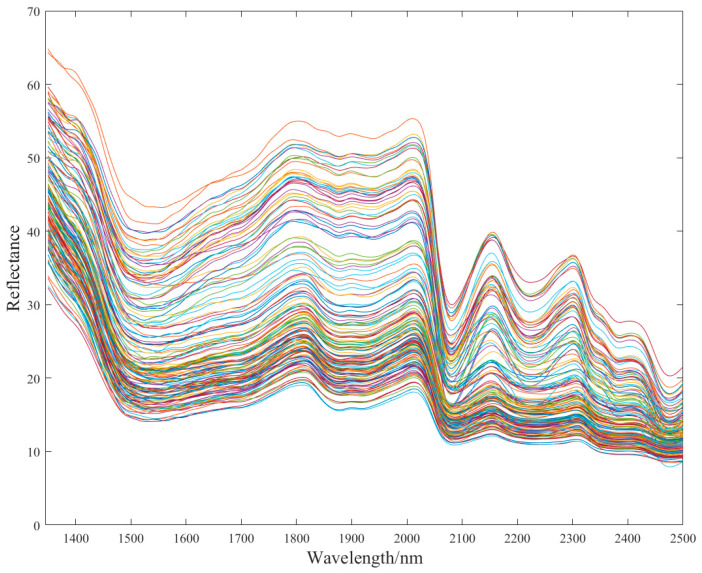
The raw near-infrared (NIR) spectrogram of oats.

Based on the raw near-infrared spectra of oat samples presented in Figure 1, four distinct absorption peaks were observed within the range of 1350–2500 nm, specifically located at 1795 nm, 2010 nm, 2154 nm, and 2297 nm. Previous research had shown that these absorption peaks could be correlated with different chemical components. The peak at 1795 nm might be associated with the first overtone of the C-H groups in the fatty acids present in oats. The absorption peaks at 2010 nm and 2154 nm might be related to the stretching and combination vibrations of N-H and C-O bonds in proteins, or the third overtone of C-O-O stretching in polysaccharides [46,47]. Lastly, the peak at 2297 nm might be associated with O-H bending, starch, and C-H stretching in fats or aliphatic C-H bonds [48].

### 3.3. Results of Different Preprocessing Methods for Total Starch, Protein, β-Glucan, and Fat in Oats

Table 2 presented the results of applying eight different preprocessing methods to the four oat indicators. The optimal preprocessing method for total starch was SD, with an *R*_c_^2^ of 0.951, *RMSEC* of 0.955, *R*_p_^2^ of 0.765, and *RMSEP* of 3.199. For protein, the MSC preprocessing method produced an *R*_c_^2^ of 0.949, *RMSEC* of 0.618, *R*_p_^2^ of 0.768, and *RMSEP* of 1.301. Regarding β-glucan, BC has been proven to be the most advantageous, with an *R*_c_^2^ of 0.958, *RMSEC* of 0.125, *R*_p_^2^ of 0.732, and *RMSEP* of 0.535. Lastly, SS had been identified as the optimal preprocessing method for fat, with an *R*_c_^2^ of 0.949, *RMSEC* of 0.434, *R*_p_^2^ of 0.847, and *RMSEP* of 0.941.

### 3.4. Results of Different Characteristic Wavelength Selection Algorithms for Total Starch, Protein, β-Glucan, and Fat in Oats

**Figure 2:**
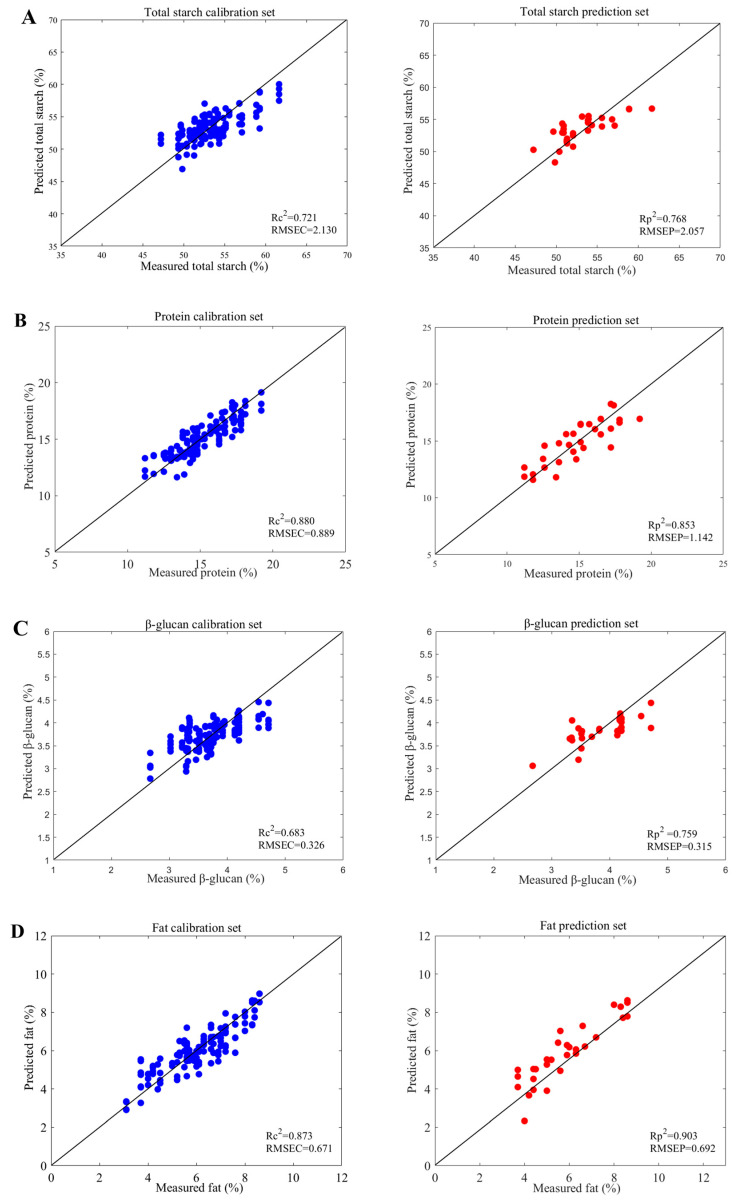
Predicted results for total starch (**A**), protein (**B**), β-glucan (**C**), and fat (**D**).

To ensure the accuracy and robustness of the model, this study applied CARS, SPA, UVE, and LAR feature wavelength selection algorithms to the four oat indicators: total starch, protein, β-glucan, and fat. The results are shown in Table 3. After SD-SPA preprocessing, the modeling data for total starch reached the optimal level, with an *R*_c_^2^ of 0.721, *RMSEC* of 2.130, *R*_p_^2^ of 0.768, and *RMSEP* of 2.057. After MS-CARS preprocessing, the modeling data for protein reached the optimal level, with an *R*_c_^2^ of 0.880, *RMSEC* of 0.889, *R_p_^2^* of 0.853, and *RMSEP* of 1.142. After BC-SPA preprocessing, the modeling data for β-glucan reached the optimal level, with an *R*_c_^2^ of 0.683, *RMSEC* of 0.326, *R*_p_^2^ of 0.759, and *RMSEP* of 0.315. After SS-SPA preprocessing, the modeling data for fat reached the optimal level, with an *R*_c_^2^ of 0.873, *RMSEC* of 0.671, *R*_p_^2^ of 0.903, and *RMSEP* of 0.692. Figure 2 demonstrated the prediction results for total starch, protein, β-glucan, and fat with the optimal models.

### 3.5. Characteristic Wavelength Analysis

**Figure 3:**
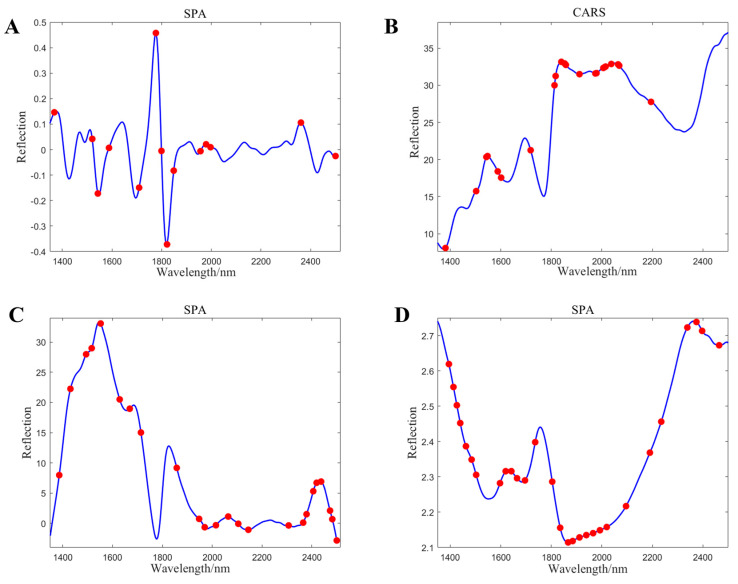
The feature wavelengths of total starch (**A**), protein (**B**), β-glucan (**C**), and fat (**D**).

In Figure 2, the wavelength graphs are depicted to illustrate the optimal performance of the total starch, protein, β-glucan, and fat models after being processed by different algorithms. Figure 3A shows the 16 selected wavelength points for total starch after the SPA, which were 1368, 1520, 1543, 1588, 1709, 1776, 1799, 1821, 1848, 1956, 1978, 1996, 1997, 1998, 2360, and 2500 nm. Figure 3B displayed the 21 optimal wavelength points for protein selected by the CARS algorithm, namely 1381, 1502, 1543, 1547, 1588, 1601, 1718, 1812, 1817, 1839, 1853, 1857, 1911, 1974, 1978, 2005, 2014, 2037, 2064, 2068, and 2194 nm. The wavelength graph for β-glucan after SPA processing is presented in Figure 3C, with the 24 selected wavelength points being 1386, 1431, 1496, 1516, 1552, 1628, 1669, 1714, 1858, 1947, 1970, 2014, 2015, 2064, 2145, 2307, 2365, 2379, 2406, 2419, 2437, 2473, 2482, and 2500 nm. Finally, Figure 3D showcases the wavelength graph for fat after SPA processing, with the 31 selected wavelength points being 1395, 1413, 1426, 1430, 1462, 1485, 1503, 1597, 1619, 1642, 1664, 1696, 1736, 1804, 1833, 1834, 1835, 1867, 1884, 1911, 1938, 1965, 1992, 2019, 2095, 2190, 2234, 2338, 2374, 2397, and 2464 nm.

## 4. Discussion

Oats possess a wealth of nutrients, including protein, fat, and β-glucan, which contribute to their esteemed reputation for promoting cholesterol reduction, regulating blood sugar levels, and facilitating digestion [49,50]. Precisely determining the nutrient composition of oats assumes paramount importance as it enables the evaluation of their nutritional value, facilitates the development of related products, and optimizes production processes [51]. Nonetheless, conventional detection methods are often time-consuming, resource-intensive, and destructive, impeding the progress of the oat industry. Fortunately, the advent of NIR non-destructive testing, driven by technological advancements and the growing demand for healthier alternatives, presents a rapid, accurate, reliable, convenient, and sustainable solution [22]. This detection technique employs non-invasive optical methods for obtaining the necessary information, thereby effectively circumventing any potential damage or contamination to the samples. Furthermore, it is characterized by its time-saving and efficient nature, enabling rapid data acquisition and significantly enhancing testing efficacy. Moreover, the technique demonstrates an elevated sensitivity, enabling the detection of subtle alterations within the samples and ensuring precise test outcomes. These extensive benefits have resulted in its widespread adoption within the realm of agricultural products. For instance, Zheng et al. (2024) effectively developed models for quantifying moisture and protein content in corn through the integration of near-infrared spectroscopy, chemometrics, and Partial Least Squares regression (PLSR) [52]. Similarly, Han et al. (2017) accomplished the establishment of prediction models for total phenols and free p-coumaric acid content in barley seeds by employing non-destructive detection with near-infrared spectroscopy [53]. Furthermore, Zhu et al. (2024) notably enhanced the precision of zearalenone content determination in wheat through the utilization of FT-NIR along with a feature wavelength optimization algorithm [27]. However, the application of near-infrared technology in oat research is still relatively limited. The objective of this study was to employ this technology to detect various indicators of oats, enabling a rapid and accurate assessment of the most critical nutritional components in oats. This method has the potential to more effectively evaluate the nutritional value of oats, offer consumers high-quality products, and optimize the production process. As a result, it creates a promising outlook for the oat industry’s advancement and expansion.

Table 1 presents the content range, mean values, and coefficient of variation for the calibration and prediction sets of four indicators in the oat samples: total starch, protein, β-glucan, and fat. The results demonstrate that the content ranges of samples in the prediction set aligned with those in the calibration set. Moreover, both the mean values and coefficients of variation for the calibration and prediction sets fell within reasonable ranges, indicating the reasonable and feasible partitioning of the dataset in this study. This provided a solid foundation for establishing reliable prediction models.

To address issues such as baseline drift and noise interference, this study employed eight different preprocessing methods to handle the raw spectral data for total starch, protein, β-glucan, and fat. Table 2 demonstrates the significant improvements in the modeling outcomes after preprocessing. The SD method exhibited the best performance for total starch, with *R*_p_^2^ increasing from 0.321 to 0.765 and *RMSEP* decreasing from 3.418 to 3.199. This highlighted the effectiveness of the SD method in eliminating baseline drift, suppressing noise, and reducing the influence of interfering factors in the total starch spectra, thus extracting valuable information [54]. For protein analysis, the MSC method enhanced the modeling results by eliminating disturbance information caused by scattering and enhancing the absorption information relevant to the experiment [55]. *R*_p_^2^ increased from 0.592 to 0.768, and *RMSEP* decreased from 1.349 to 1.301. The BC method yielded the best results for β-glucan, with *R*_p_^2^ increasing from 0.644 to 0.732 and *RMSEP* changing from 0.383 to 0.535. By reducing or eliminating interference from background signals in the spectra, the BC method enhanced the reliability of the analysis results [56]. Finally, for fat analysis, the SS method demonstrated optimal performance by smoothing and processing the raw spectral data, thereby reducing noise and irregularities [57]. This improved the spectral quality, with *R*_p_^2^ increasing from 0.838 to 0.847 and *RMSEP* changing from 0.900 to 0.941. Employing appropriate preprocessing methods effectively eliminated interference and enhanced the accuracy and reliability of the modeling results. It is important to note that the optimal preprocessing method might vary for different indicators, emphasizing the significance of employing targeted preprocessing methods specific to each indicator.

To further enhance model accuracy, this study employed four feature wavelength selection algorithms, namely CARS, SPA, UVE, and LAR, in addition to the optimal preprocessing methods for total starch, protein, β-glucan, and fat. Table 3 presented the results. After SD-SPA processing, the total starch results reached their optimum, with *R*_p_^2^ increasing from 0.765 to 0.768 and *RMSEP* decreasing from 3.199 to 2.057. For protein, the MSC-CARS processing yielded the best results, with *R*_p_^2^ increasing from 0.768 to 0.853 and *RMSEP* decreasing from 1.301 to 1.142. The BC-SPA processing resulted in the optimal β-glucan results, with *R*_p_^2^ increasing from 0.732 to 0.759 and *RMSEP* decreasing from 0.535 to 0.315. Finally, the SS-SPA processing led to the best fat results, with *R*_p_^2^ increasing from 0.847 to 0.903 and *RMSEP* decreasing from 0.941 to 0.692. These results demonstrated that employing feature wavelength selection algorithms could further enhance prediction accuracy, reduce model complexity, and decrease data processing time and computational resource consumption. This played a crucial role in the rapid and non-destructive detection of oat indicators. The progressive evolution of technology brought about the emergence of near-infrared non-destructive testing as an indispensable tool for quality control in oats. This state-of-the-art technology not only improved the efficiency and accuracy of oat processing but also offered a more efficient and dependable testing method, thereby contributing to the advancement of the grain industry. Consequently, conducting further research and expanding the application of near-infrared technology in oat quality control and assessment hold immense significance and offer extensive prospects for implementation.

## 5. Conclusions

By combining NIR with various preprocessing methods and feature wavelength selection algorithms, this study successfully achieved the rapid and non-destructive detection of total starch, protein, β-glucan, and fat content in oat grains. After preprocessing, the correlation between NIR and the four indicators’ content significantly improved. The different preprocessing methods showed varying effectiveness for each indicator: for total starch, the SD method performed the best (*R*_p_^2^ = 0.765, *RMSEP* = 3.199), for protein, the MSC method showed an excellent performance (*R*_p_^2^ = 0.768, *RMSEP* = 1.301), for β-glucan, the BC method yielded the best results (*R*_p_^2^ = 0.732, *RMSEP* = 0.535), and for fat, the SS method performed the best (*R*_p_^2^ = 0.847, *RMSEP* = 0.941). On the basis of the optimal preprocessing methods, feature wavelength selection was conducted. After SD-SPA processing, the total starch results reached their peak with an *R*_p_^2^ of 0.768 and *RMSEP* of 2.057. After MSC-CARS processing, the protein results achieved their best result with an *R*_p_^2^ of 0.853 and *RMSEP* of 1.142. After BC-SPA processing, the β-glucan results reached their optimum with an *R*_p_^2^ of 0.759 and *RMSEP* of 0.315. Finally, after SS-SPA processing, the fat results achieved their peak with an *R*_p_^2^ of 0.903 and *RMSEP* of 0.692. These results demonstrated that, by employing feature wavelength selection algorithms, the predictive accuracy of the models could be further enhanced, model complexity could be reduced, and data processing time and computational resources could be saved, playing a vital role in the rapid and non-destructive detection of oat indicators. Currently, the application of near-infrared spectroscopy technology in the detection of oat quality is relatively scarce in the market. However, this study fills a crucial gap in this field and demonstrates the practical value of near-infrared spectroscopy in oat quality analysis. By employing appropriate pretreatment and feature selection methods, near-infrared spectroscopy can provide an efficient and non-destructive approach to detection, offering a viable solution for quality control and testing in the oat industry. It can assist oat producers in selecting high-quality oat varieties, thereby enhancing product competitiveness and market share. Additionally, it can guide the optimization of oat processing techniques, leading to improved product processing efficiency and quality stability. Moreover, it establishes a scientific foundation for the quality assessment and monitoring of oat products, ensuring adherence to relevant quality standards and requirements. Furthermore, this study holds considerable implications for research and practical applications in related fields. The potential application prospects of near-infrared spectroscopy technology in agricultural product detection are extensive, providing novel solutions and methods for quality control and testing across various agricultural products. Therefore, the findings of this study play a pivotal role in advancing the application and development of near-infrared spectroscopy technology in the agricultural sector.

## Figures and Tables

**Table 1:** Distributions of the calibration and prediction set reference values.

Variables	Dataset	Statistical Parameters			
		Samples	Range	Mean	Sd
Total starch (%)	Calibration	119	47.22–61.66	53.41	3.16
	Prediction	30	47.22–58.88	53.18	2.90
Protein (%)	Calibration	119	11.20–19.20	14.97	2.00
	Prediction	30	12.60–17.80	15.64	1.57
β-glucan (%)	Calibration	119	2.67–4.71	3.74	0.45
	Prediction	30	2.67–4.71	3.80	0.50
Fat (%)	Calibration	119	3.10–8.60	5.94	1.38
	Prediction	30	3.70–8.60	6.02	1.60

**Table 2:** Models’ results for total starch, amylose starch, protein, β-glucan, total phenols, and fat with different spectral pretreatment methods.

Parameters	Pretreatment Method	*R*_c_^2^	*RMSEC*	*R*_p_^2^	*RMSEP*
Total starch	Raw	0.950	0.968	0.321	3.418
	SNV	0.952	0.924	0.517	3.861
	MSC	0.950	0.938	0.490	3.878
	Nor	0.955	0.912	0.404	4.407
	DE	0.954	0.892	0.525	3.475
	FD	0.953	0.909	0.494	3.551
	SD	0.951	0.955	0.765	3.199
	BC	0.951	0.944	0.595	3.817
	SS	0.951	0.972	0.455	3.999
Protein	Raw	0.949	0.630	0.592	1.349
	SNV	0.950	0.589	0.762	1.869
	MSC	0.949	0.618	0.768	1.301
	Nor	0.950	0.583	0.763	1.821
	DE	0.949	0.618	0.553	1.555
	FD	0.949	0.612	0.653	1.683
	SD	0.949	0.625	0.622	1.351
	BC	0.949	0.631	0.570	1.413
	SS	0.949	0.634	0.621	1.362
β-glucan	Raw	0.955	0.131	0.644	0.383
	SNV	0.953	0.134	0.590	0.458
	MSC	0.950	0.138	0.560	0.471
	Nor	0.954	0.130	0.517	0.130
	DE	0.949	0.137	0.420	0.544
	FD	0.955	0.136	0.231	0.552
	SD	0.952	0.139	0.458	0.476
	BC	0.958	0.125	0.732	0.535
	SS	0.950	0.138	0.714	0.544
Fat	Raw	0.952	0.422	0.838	0.900
	SNV	0.949	0.435	0.782	0.990
	MSC	0.949	0.429	0.802	1.006
	Nor	0.950	0.435	0.810	0.913
	DE	0.951	0.429	0.777	0.999
	FD	0.950	0.423	0.762	1.070
	SD	0.949	0.426	0.756	1.117
	BC	0.949	0.433	0.730	1.095
	SS	0.949	0.434	0.847	0.941

**Table 3:** Results of PLS models with multiple quality parameters for oats based on different feature wavelength selection algorithms.

Parameters	Method	*R*_c_^2^	*RMSEC*	*R*_p_^2^	*RMSEP*
Total starch	SD-CARS	0.755	2.017	0.702	2.322
	SD-SPA	0.721	2.130	0.768	2.057
	SD-UVE	0.984	0.553	0.383	9.998
	SD-LAR	0.978	0.637	0.658	5.437
Protein	MSC-CARS	0.880	0.889	0.853	1.142
	MSC-SPA	0.842	1.011	0.844	1.165
	MSC-UVE	0.978	0.395	0.715	2.485
	MSC-LAR	0.989	0.281	0.709	2.487
β-glucan	BC-CARS	0.813	0.252	0.422	0.464
	BC-SPA	0.683	0.326	0.759	0.315
	BC-UVE	0.767	0.278	0.478	0.440
	BC-LAR	0.984	0.078	0.685	0.740
Fat	SS-CARS	0.884	0.781	0.856	0.831
	SS-SPA	0.873	0.671	0.903	0.692
	SS-UVE	0.978	0.285	0.728	1.265
	SS-LAR	0.984	0.244	0.777	1.095

## Data Availability

The original contributions presented in the study are included in the article, further inquiries can be directed to the corresponding author.
